# Supplementary material for: Relationship between higher education teachers’ affect and their psychological adjustment to online teaching during the COVID-19 pandemic: an application of latent profile analysis
Source: PeerJ. 2021 Nov 3;9:e12432. doi: 10.7717/peerj.12432 (PMC8571955; doi:10.7717/peerj.12432)
Supplement: Supplemental Information 2 [file peerj-09-12432-s002.docx]

**高校教师线上教学心理适应调查量表**

1. 我相信自己能胜任网络教学，只要我努力就能上好每一堂网课。

① 从未 ② 很少 ③ 有时 ④ 经常 ⑤ 总是

2. 线上网络教学时，各种网络故障（卡顿、掉线等）会使我思路慌乱、沮丧和不安。

① 从未 ② 很少 ③ 有时 ④ 经常 ⑤ 总是

3. 线上网络教学期间，我会主动与其他老师分享和交流自己的经验，彼此支持与互助。

① 从未 ② 很少 ③ 有时 ④ 经常 ⑤ 总是

4. 线上网络授课时，由于不能与学生面对面交流，我会常因担心学生没听懂而焦虑和不安。

① 从未 ② 很少 ③ 有时 ④ 经常 ⑤ 总是

5. 我对学习与探索线上网络教学这门新技能充满热情与好奇，在挑战自己的同时突破自我。

① 从未 ② 很少 ③ 有时 ④ 经常 ⑤ 总是

6. 每次网络授课我都精力充沛，体验着当“主播”的激情与新鲜。

① 从未 ② 很少 ③ 有时 ④ 经常 ⑤ 总是

7. 线上网络授课时，我会由于无法掌控学生在另一端的参与程度而忧虑与无奈。

① 从未 ② 很少 ③ 有时 ④ 经常 ⑤ 总是

8. 如果第二天有自己的网课，我会担心线上网络授课效果不好、质量不高而睡不好觉。

① 从未 ② 很少 ③ 有时 ④ 经常 ⑤ 总是

9. 进行网络教学直播时，我会觉得不自在，比平常容易紧张或着急。

① 从未 ② 很少 ③ 有时 ④ 经常 ⑤ 总是

10. 线上网络教学需要更多的时间和精力备课，因而每当完成一堂线上网络教学课后，在轻松的同时我会感到体弱和疲惫。

① 从未 ② 很少 ③ 有时 ④ 经常 ⑤ 总是

11. 由于在线上网络教学的同时还要照顾小孩而感到无助与烦闷。

① 从未 ② 很少 ③ 有时 ④ 经常 ⑤ 总是

12. 在不熟悉网络教学平台和直播软件操作时，我常处于焦灼状态。

① 从未 ② 很少 ③ 有时 ④ 经常 ⑤ 总是

13. 线上网络授课，我会心情愉悦充满爱与期待去与学生保持良好沟通。

① 从未 ② 很少 ③ 有时 ④ 经常 ⑤ 总是

14. 每一堂线上网络授课，我会温馨关照那些因没有网络、没有流量或网络信号差等，不能正常参与线上课堂的学生，为他们制定一人一案的学习安排。

① 从未 ② 很少 ③ 有时 ④ 经常 ⑤ 总是
