# Supplementary material for: Relationship between higher education teachers’ affect and their psychological adjustment to online teaching during the COVID-19 pandemic: an application of latent profile analysis
Source: PeerJ. 2021 Nov 3;9:e12432. doi: 10.7717/peerj.12432 (PMC8571955; doi:10.7717/peerj.12432)
Supplement: Supplemental Information 3 [file peerj-09-12432-s003.docx]

**the Psychological Adjustment to Online Teaching Scale**

1. I believe that I can be qualified for online teaching. As long as I work hard, I can take every online class well.

① Never ② Rarely ③ Sometimes ④ Often ⑤ Always

2. During online teaching, various network failures (stuttering, disconnection, etc.) make my mind flustered, frustrated and uneasy.

① Never ② Rarely ③ Sometimes ④ Often ⑤ Always

3. During the online teaching, I will actively share and exchange my experience with other teachers, and support and help each other.

① Never ② Rarely ③ Sometimes ④ Often ⑤ Always

4. When I teach online, because I cannot communicate with students face-to-face, I often feel anxious and uneasy because I am worried that the students will not understand.

① Never ② Rarely ③ Sometimes ④ Often ⑤ Always

5. I am full of enthusiasm and curiosity about learning and exploring the new skill of online teaching, and I am challenging myself while breaking through myself.

① Never ② Rarely ③ Sometimes ④ Often ⑤ Always

6. I am energetic every time I teach online, and I experience the passion and freshness of being an "anchor".

① Never ② Rarely ③ Sometimes ④ Often ⑤ Always

7. When teaching online, I feel worried and helpless because I cannot control the degree of student participation on the other end.

① Never ② Rarely ③ Sometimes ④ Often ⑤ Always

8. If I had my own online class the next day, I would worry that the online class would not be effective, the quality would be low, and I would not sleep well.

① Never ② Rarely ③ Sometimes ④ Often ⑤ Always

9. When conducting online teaching live broadcasts, I feel uncomfortable and get nervous or anxious more than usual.

① Never ② Rarely ③ Sometimes ④ Often ⑤ Always

10. Online teaching requires more time and energy to prepare lessons, so after completing an online teaching class, I feel weak and tired while relaxing.

① Never ② Rarely ③ Sometimes ④ Often ⑤ Always

11. I feel helpless and bored because I have to take care of my children while teaching online.

① Never ② Rarely ③ Sometimes ④ Often ⑤ Always

12. When I am not familiar with the operation of online teaching platforms and live broadcast software, I am often in a state of anxiety.

① Never ② Rarely ③ Sometimes ④ Often ⑤ Always

13. When I teach online, I will be happy and full of love and expectation to maintain good communication with students.

① Never ② Rarely ③ Sometimes ④ Often ⑤ Always

14. For each online class, I will warmly take care of those students who are unable to participate in the online class due to lack of internet, no traffic or poor network signal, etc., and make a one-person-one-case study arrangement for them.

① Never ② Rarely ③ Sometimes ④ Often ⑤ Always
